# CPADS-30: Mastering the first 30 seconds of adult cardiac arrest resuscitation

**DOI:** 10.1016/j.ajem.2025.06.056

**Published:** 2025-06-23

**Authors:** James S. Ford, Atul Malhotra, Alex K. Pearce, Gabriel Wardi

**Affiliations:** a Department of Emergency Medicine, University of California San Diego, San Diego, CA, United States of America; b Department of Medicine, University of California San Diego, San Diego, CA, United States of America

**Keywords:** Cardiac arrest, Resuscitation, Code leadership

## Abstract

**Background:**

Advanced Cardiovascular Life Support (ACLS) knowledge and skills retention is poor among clinicians. Deviations from ACLS guidelines are common and are associated with worse outcomes and less experienced code leaders often feel unprepared to lead resuscitations.

**Objective:**

To develop a schematic for assisting code leaders in managing the initial phase of cardiac arrest resuscitations.

**Methods:**

We reviewed the medical literature evaluating the effectiveness and timing of ACLS interventions in adult cardiac arrest, with a focus on identifying tasks most strongly associated with patient-centered outcomes and those most closely aligned with existing ACLS protocols. Four clinical content experts assessed the literature and independently ranked tasks to be included in the mnemonic; the final ranked list was approved by consensus discussion. We then incorporated our ranked list into a mnemonic using principles of cognitive load theory, including chunking, serial recall, and the primacy effect.

**Results:**

We identified five early interventions with strong evidence supporting their impact on outcomes. These interventions form the core of **“CPADS-30”**, a simplified mnemonic designed to facilitate memory recall and facilitate task delegation in the first 30 s of cardiac arrest resuscitation. These tasks include **C** (Chest compressions), **P** (Pad [defibrillator] placement), **A** (Access [intravenous/intraosseous]), **D** (Drug administration) and **S** (Scribe assignment).

**Conclusion:**

**“CPADS-30”** distills down critical *early* actions of cardiac arrest management into a discreet, digestible schematic that complements the complete ACLS algorithm. We hope **“CPADS-30”** will prove useful to trainees and providers of all levels of training in various practice environments.

## 1. Background

Despite millions of dollars invested in research efforts to improve patient outcomes, cardiac arrest survival remains poor [1]. The American Heart Association (AHA) Advanced Cardiovascular Life Support (ACLS) treatment algorithms remain the standard-of-care in the resuscitation of cardiac arrest, and ACLS certification is required for many providers who work in acute care settings [2]. Despite efforts to sustain provider competence, ACLS knowledge and skills quickly extinguish, with skill competency dropping to less than 40 % in physicians and less than 14 % in nurses, at one year [3–5]. In cardiac arrest resuscitations, the code leader must delegate tasks, vocalize critical actions and summaries, consider differential diagnoses and execute management decisions. The code leader must juggle these responsibilities under extreme time constraints, while facing a barrage of sensory inputs, which may deleteriously impact code leadership. This high-stress, high-acuity environment places a substantial cognitive burden on the provider, which is known to compromise neurocognitive performance [6–9]. Given poor knowledge retention and the high cognitive load associated with managing cardiac arrest, it is unsurprising that less experienced code leaders often feel unprepared to lead resuscitations [10–12].

Deviations from ACLS guidelines are common and are associated with poor outcomes [13–15]. A study of simulated cardiac arrest resuscitations found that the average time to beginning chest compressions after diagnosis of cardiac arrest was 37 s [16]. In this study, as many as 25 unique different actions were initiated by team members prior to the initiation of chest compressions [16]. In a real-world study of cardiac arrest, only 25 % of patients had defibrillation pads placed after 5 min of resuscitation [17]. Studies in adults have shown that delayed defibrillation (greater than 2 min from cardiac arrest) occurs in almost one-third of cases with shockable rhythms (pulseless ventricular tachycardia, pVT; ventricular fibrillation, VF) [18,19]. These data suggest that when code leaders confront the challenging environment of a cardiac arrest resuscitation, cognitive performance deteriorates and critical actions may be delayed.

In qualitative studies of cardiac resuscitation, poor task assignment is frequently cited as an opportunity for improvement [20]. In real-world clinical settings, tasks are often performed in parallel by experienced team members without prompting from the code leader. However, in smaller or less-experienced resuscitation teams, the *order* in which tasks are assigned and completed is important. Mental checklists, such as short memory mnemonics, are known to reduce error and decrease cognitive load, particularly in high acuity encounters [6–8,21] While ACLS provides a comprehensive algorithm for cardiac arrest resuscitations, code leaders may benefit from a simplified opening playbook that reduces cognitive burden and helps prioritize tasks and interventions that have been shown to meaningfully affect patient-centered outcomes.

In this manuscript, we describe “CPADS-30,” a novel, simple mnemonic developed to assist resuscitation code leaders in accomplishing a series of critical tasks in the first 30 s of adult cardiac resuscitation (Table 1).

## 2. Clinical content expert assessment

Four clinical content experts in adult cardiac arrest resuscitation independently reviewed the literature for the effectiveness and timing of ACLS interventions in adult cardiac arrest, with a focus on identifying tasks most strongly associated with patient-centered outcomes and those most closely aligned with existing ACLS protocols. Each content expert was asked to generate a list of five critical tasks to be included in the mnemonic. Next, clinical experts met to discuss their lists, and the final ranked list was approved by consensus discussion. We then incorporated our ranked list into a mnemonic using principles of cognitive load theory, including chunking, serial recall, and the primacy effect. The final mnemonic, which aimed to assign five critical tasks in the first 30 s of cardiac arrest resuscitation, was named “CPADS-30.” These tasks are described further below.

## 3. Rationale and evidence underpinning “CPADS-30” interventions

1. ***“C” – Chest compressions:*** Initiate high-quality cardiopulmonary resuscitation (CPR).
  - **Rationale:** High-quality CPR is the first critical task because it is the most effective intervention for immediately restoring cardiac output and end-organ perfusion.
  - **Evidence**: Studies have consistently demonstrated that CPR improves outcomes in out-of-hospital cardiac arrest (OHCA) and in-hospital cardiac arrest (IHCA). Early CPR is associated with earlier return of spontaneous circulation (ROSC), improved survival to discharge and favorable neurological outcomes [22,23].
2. **“P” *– Pads:*** Apply defibrillator pads to the patient.
  - **Rationale:** This is the second critical task because it is essential for assessing the rhythm during the first pulse check and delivering defibrillation for shockable rhythms (pVT/VF).
  - **Evidence:** Early defibrillation is associated with improved survival and each minute delay in defibrillation decreases the likelihood of achieving return of spontaneous circulation (ROSC) by 19 % [18,19,24]. Delays in defibrillation are also associated with a lower probability of surviving to hospital discharge in IHCA and lower functional survival in OHCA [19,25]. Hospitals in the highest-performing quartile for time-to-defibrillation have lower IHCA mortality compared to hospitals in the lowest-performing quartiles [26].
3. **“A” – *Access*:** Placement of an intravenous (IV) or intraosseous (IO) catheter.
  - **Rationale:** This is the third critical task. Obtaining IV/IO access is essential for administering medications that are part of the ACLS cardiac arrest algorithm.
  - **Evidence:** Two recent randomized controlled trials studies compared IV to IO placement in OHCA and found no difference in time to catheter placement, 30-day survival or neurologically-intact survival [27,28]. To our knowledge, no published studies have assessed the association between time-to-catheter placement and cardiac arrest mortality.
4. ***“D” – Drugs:*** Assign a team member to administer medications.
  - **Rationale**: This is the fourth critical task, as IV/IO access must be established before medications can be delivered.
  - **Evidence**: Epinephrine has been shown to improve rates of ROSC; however, its effect on survival to hospital discharge and overall survival remains inconclusive, and data show it may worsen survival with favorable neurologic function [29,30]. Previous studies have shown no association between anti-arrhythmic drugs (lidocaine and amiodarone) in shockable rhythms (pVT/VF) and survival [31,32]. While data underpinning the benefit of medication adjuncts in cardiac arrest are less robust, they remain an integral part of ACLS cardiac arrest management and may be beneficial depending on the underlying etiology of cardiac arrest.
5. ***“S” - Scribe:*** Assign a team member to keep time and document code details.
  - **Rationale**: This is the fifth critical task to assign because real-time documentation and timekeeping are essential for ensuring patients receive interventions at the intervals established in the ACLS algorithm. Additionally, code timelines are helpful important for debriefing and quality improvement (QI). This role can be fulfilled by a nurse, patient care tech, junior provider, or other available team member.
  - **Evidence**: The impact of the resuscitation scribe on patient outcomes has been poorly studied. However, assigning this task is frequently overlooked and unassigned, and may impact outcomes indirectly, by helping with cognitively offloading the resuscitation leader, and my helping improve future resuscitation efforts through QI processes [20].

## 4. Cognitive theory underlying “CPADS-30” mnemonic development

We designed this mnemonic using principles of cognitive load theory (CLT), with the goal of reducing clinician cognitive burden and facilitating rapid memory recall.

- ***Information “Chunking”:*** “Chunking” is the mechanism by which the brain groups related pieces of information to improve recall [33]. Research suggests that individuals can only hold about five items in their working memory (WM) [34]. Thus, we deliberately included just five tasks in CPADS-30 to minimize burden on the WM.
- ***Serial Recall and Memory Chains:*** Serial recall is the process by which “chunks” of information are recalled in an ordered sequence (memory chains), whereby recalling an initial unit of information primes the activation of the next information unit, and so on [35]. The alphabet song is a classic example of this type of recall mechanism.
- ***Schema Formation:*** With repeated practice, mnemonics such as CPADS-30, integrate underlying “chunks” of information into ordered memory chains and store them in organized mental schema. Schema provide important context for learning and encode both declarative (“what”) and procedural (“how”) information, which directly links information recall to action [36].
- ***Written Semantic Cues:*** The first two letters in CPADS-30 are “C” and “P”, which nicely correspond to the first two letters in the first critical task: CPR. Similarly, the second written syllable “PADS” corresponds to the second critical task: placement of pads on the patient. These “hidden” written cues provide additional semantic memory triggers.
- ***Primacy Effect and Task Prioritization:*** The primacy effect refers to the tendency to recall information units at the beginning of a sequence [37]. The two interventions with the most robust effect on mortality, CPR and defibrillation, are listed first and second in the CPADS-30 mnemonic, to help prioritize the most important tasks.
- ***Reduction of Intrinsic Cognitive Load:*** During a resuscitation, the code leader is confronted with multiple competing stimuli and decision forks. This situation can be cognitively overwhelming and distract from completing critical tasks. CPADS-30 incorporates various principles from CLT that aim to minimize the mental effort required to process and execute a complex series of tasks (the entire ACLS algorithm) while managing a stream of competing stimuli.

## 5. Considerations for clinical implementation and future directions

In summary, CPADS-30 is a simplified mental framework and memory mnemonic designed to assist code leaders during the first 30 s of cardiac resuscitation. CPADS-30 is not intended to be a comprehensive roadmap for cardiac resuscitation. On the contrary, this mnemonic intends to distill down critical early actions of cardiac arrest management into a discreet, digestible schematic that complements the complete ACLS algorithm. Notably, this mnemonic is not intended for use in children, as pediatric cardiac arrest resuscitations have special considerations. We hope that this tool can be utilized by pre-hospital providers and clinicians in all hospital settings. While we expect that CPADS-30 will prove most useful to trainees and early-career providers, we believe that it will bring value to clinicians regardless of level of training. While this tool was developed by consensus discussion from four clinical content experts in cardiac arrest resuscitation, future refinements of this mnemonic would benefit from more objective development methods (i.e. Delphi technique) [38]. Future studies will assess whether CPADS-30 can improve important process measures such as time-to-defibrillation or important outcome measures such as ROSC or survival to hospital discharge.

## Figures and Tables

**Table 1:** CPADS-30 Mnemonic for Adult Cardiac Arrest Resuscitation.

	Task	Explanation

C	Chest compressions	Assign first and second CPR rounds
P	Pads (Defibrillator)	Place pads, hook up to defibrillator
A	Access (IV/IO)	Establish IV/IO access
D	Drugs	Pull and be ready to give medications
S	Scribe	Documentation and time keeping
30	Thirty seconds	Prioritize above tasks

CPR, cardiopulmonary resuscitation. IO, intraosseous line. IV, intravenous line.
